# Tei Index Is a Useful Adjunctive Tool in the Diagnostic Workup of Patients with Acute Myocarditis

**DOI:** 10.3390/jcdd9080283

**Published:** 2022-08-22

**Authors:** Moritz Mirna, Lukas Schmutzler, Fabian Vogl, Albert Topf, Uta C. Hoppe, Michael Lichtenauer

**Affiliations:** Department of Internal Medicine II, Division of Cardiology, Paracelsus Medical University of Salzburg, 5020 Salzburg, Austria

**Keywords:** myocarditis, acute coronary syndrome, ACS, echocardiography, Tei index, TI, ejection fraction, diastolic dysfunction

## Abstract

Background: Tei index (TI) is a combined myocardial performance index, which was found to be more sensitive for overall cardiac dysfunction than systolic or diastolic parameters alone. Currently, there is only limited evidence for this measure in the context of myocarditis. Thus, TI could add additional benefits to conventional diagnostic workup. Methods: TI of patients with myocarditis (*n* = 40), acute coronary syndrome (*n* = 29) and controls (*n* = 50) was retrospectively analyzed concerning its discriminatory ability for myocarditis. Results: TI was most pathological in patients with myocarditis (median 0.41 vs. 0.35 vs. 0.31, *p <* 0.0001). Its discriminatory ability was better than that of EF or E/e’ (AUCs: TI: 0.71, *p* < 0.0001; EF: 0.57, *p* = 0.112; E/e’: 0.64, *p* = 0.983), which was also verified in logistic regression analysis (B(SE) = 0.81(0.23), *p* = 0.0004). The association of TI with myocarditis remained significant even after correction for confounders in propensity score weighted analysis. Conclusions: The TI showed a better discriminatory ability for myocarditis than conventional echocardiographic parameters. Since TI is easily conducted, it might be a helpful adjunctive tool to supplement conventional diagnostic modalities in patients with suspected myocarditis.

## 1. Introduction

By accounting for approximately 10% of all non-injury-related visits, acute chest pain constitutes one of the most frequent complaints of patients presenting to the emergency department [1]. Of the possible causes for this symptom, myocarditis represents a comparatively infrequent, yet important, disease entity. Since myocarditis can be associated with formidable adverse outcomes, a timely diagnostic workup and initiation of adequate treatment are required in affected patients [2,3].

In clinical practice, the diagnostic workup of patients with chest pain usually includes an electrocardiogram (ECG), biomarkers for myocardiocytolysis and transthoracic echocardiography (TTE) [1,4,5]. However, several of these diagnostic applications are not specific for myocarditis [6]. Notably, this especially applies to TTE, which has become an indispensable diagnostic application in the initial evaluation of patients with chest pain. As such, acute myocarditis can be associated with a variety of echocardiographic findings, ranging from marked left ventricular (LV) hypertrophy to LV dilatation with severely impaired systolic function [7,8,9]. Notably, the latter also constitutes the predominant echocardiographic finding in patients with acute coronary syndrome (ACS), one of the major differential diagnoses to myocarditis. In addition to the aforementioned findings, patients with myocarditis can present with an apparently normal conventional TTE exam [10,11,12], which poses additional challenges in the diagnostic workup of affected patients. Since conventional TTE frequently offers limited diagnostic benefits in the evaluation of patients with suspected myocarditis, the possible additive diagnostic value of an easily calculated echocardiographic parameter such as the Tei index (TI) could thus be of special interest for clinicians.

The TI (normal value: <0.39 ± 0.05 [13]) constitutes a myocardial performance index for the assessment of overall cardiac function, which can easily be calculated by the addition of the isovolumic contraction time to the isovolumic relaxation time divided by the ejection time [14,15]. Interestingly, the TI was previously described as a sensitive diagnostic and prognostic parameter in several diseases, including heart failure [16], LV hypertrophy [17,18] and chronic coronary syndrome [19].

However, evidence on the diagnostic implications of the TI in myocarditis is comparatively scarce, especially in comparison to healthy individuals and patients with ACS. Because of the often apparently normal TTE exam of patients presenting with myocarditis, this easily calculated index could add valuable information for the initial diagnostic workup. Thus, we sought to investigate the TI in patients with myocarditis in this study.

## 2. Materials and Methods

The study outline was reviewed and approved by the ethical review board of the state of Salzburg, Austria (EK Nr: 1181/2020) before data acquisition. The study was conducted according to the principles of Good Clinical Practice and the Declaration of Helsinki.

### 2.1. Patients

Patients with myocarditis and ACS were retrospectively identified through database search on discharge diagnoses of all patients admitted to the University Hospital of Salzburg, Austria, between the years 2009 and 2019. The International Classification of Diseases, Tenth Revision (ICD-10) code was used to classify and search for the diagnostic codes I40.0, I40.1, I40.8, I40.9, I51.4 (myocarditis) and I21.0, I21.1, I21.2, I21.3, I21.4, I21.9, I24.9 (ACS). Eligible patients were enrolled if they fulfilled the criteria for clinically suspected myocarditis [6] or the criteria for ST-segment elevation myocardial infarction (STEMI) [5] or non-ST-segment elevation myocardial infarction (NSTEMI) [20] by the European Society of Cardiology. Furthermore, patients were only enrolled if echocardiographic loops from the initial presentation to the hospital were recorded that allowed for assessment of TI. TI was assessed and calculated as previously published [15,16]. Forty patients with acute myocarditis and 29 patients with ACS were finally enrolled in the study. Fifty patients admitted to our hospital due to chest pain, in whom coronary artery disease was excluded by means of coronary angiography, served as controls.

### 2.2. Statistical Analysis

Statistical analyses were conducted with R (version 4.0.2., R Core Team (2013), R Foundation for Statistical Computing, Vienna, Austria; http://www.R-project.org/, accessed on 7 October 2021) using the packages ‘Rcmdr’, ‘ggplot2’, ‘pastecs’, ‘Hmisc’, ‘ggm’, ‘polycor’, ‘QuantPsyc’, ‘glmnet’, ‘twang’, ‘survey’, ‘stddidff’, ‘survival’ and ‘survminer’ and GraphPad Prism (Version 5.01, GraphPad Software Inc., San Diego, CA, USA). Skew and kurtosis of data were assessed visually, Shapiro–Wilk test was conducted to test for data distribution. Since distribution was not normal, metric data are depicted as median ± interquartile range (IQR) and medians were compared by Kruskal–Wallis test with Dunn’s post hoc test. Receiver operating characteristic (ROC) curve analysis was performed to assess the discriminatory ability of TI for diagnosis of myocarditis. One cut-off (cut-off #1) was calculated by means of the Youden Index [21], another one (cut-off #2) to depict maximum specificity for myocarditis in the total cohort. Binary logistic regression analysis was conducted to investigate the predictive ability of echocardiographic parameters. Prior to regression analysis, continuous data were transformed to z-scores to assure standardization. In order to account for possible imbalances in baseline covariates, propensity score weighting of the groups by Generalized Boosted Models (GBM) using the Average Treatment Effect (ATE) [22] was additionally conducted. Overlap concerns were checked for by assessment of density plots of metric data, as well as cross tabulations of nominal data. After balancing, weighted logistic regression analysis was performed for presence of myocarditis using the ‘survey’ package of R. A *p*-value of <0.05 was considered statistically significant.

## 3. Results

### 3.1. Baseline Characteristics

In total, 119 patients were enrolled in this study, 40 of whom had myocarditis (33.6%) and 29 ACS (24.4%). Patients with myocarditis were significantly younger than patients with ACS or controls (median 35 years vs. 52 years vs. 70 years, *p* < 0.0001) and traditional cardiovascular risk factors, such as diabetes mellitus, hyperlipidemia, obesity or smoking were significantly less prevalent in these patients (see Table 1).

While C-reactive protein (CRP; median 2.35 mg/dL vs. 0.20 mg/dL vs. 0.20 mg/dL, *p <* 0.0001) and high sensitivity troponin (hsTnT; myocarditis: median 375 ng/L vs. ACS: 244 ng/L vs. control: 14 ng/L, *p <* 0.0001) were significantly higher in patients with myocarditis, concentrations of serum pro brain natriuretic peptide (pBNP) were lower than in controls (see Table 1).

### 3.2. Findings of Transthoracic Echocardiography (TTE)

**Figure 1:**
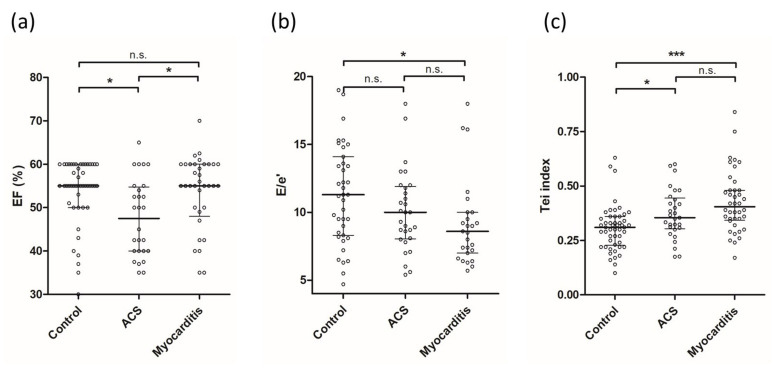
Scatter plots of (**a**) EF, (**b**) E/e‘ and (**c**) TI in patients of both groups. Depicted are median and interquartile range; *** denotes a *p*-value < 0.0001, * denotes a *p-*value < 0.05, n.s. = not significant. Abbreviations: ACS = acute coronary syndrome, EF = ejection fraction, TI = Tei index.

Table 2 depicts data from TTE upon hospital admission. Compared to patients of the other two groups, patients with myocarditis had lower values of E/e’ (myocarditis: median 8.6 vs. ACS: 10.0 vs. control: 11.3, *p* = 0.041) and significantly higher values of the TI (myocarditis: median 0.41 vs. ACS: 0.35 vs. control: 0.31, *p* < 0.0001, see Table 2 and Figure 1b,c) than patients of the other two groups.

Notably, while left ventricular (LV) systolic ejection fraction (EF) did not differ between patients with myocarditis and the controls, the discrepancy in TI between these two groups was highly significant (Figure 1a,c). Moreover, the difference in EF between ACS patients, myocarditis patients and the controls was not as pronounced as with the TI (see Figure 1a,c).

### 3.3. ROC-Analysis and Cut-Offs

**Figure 2:**
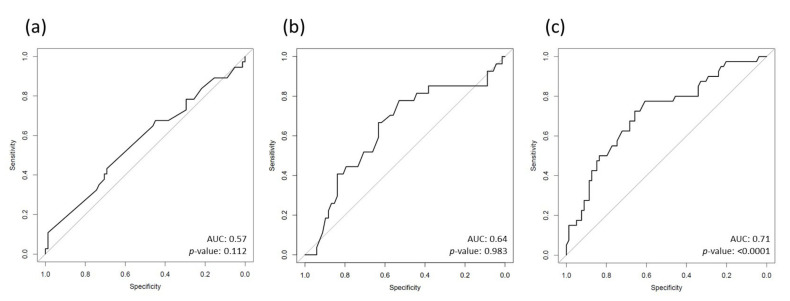
ROC-curves for diagnosis of myocarditis of (**a**) EF, (**b**) E/e‘ and (**c**) TI. Abbreviations: AUC = area under the curve, EF = ejection fraction, TI = Tei index.

ROC-analysis was conducted and AUCs were calculated for the diagnosis of myocarditis in the total study cohort. Notably, the AUC for the TI (0.71, 95%CI 0.62–0.81, *p* < 0.0001) was distinctively better than those calculated for EF (0.57, 95%CI 0.46–0.68, *p* = 0.112) or E/e’ (0.64, 95%CI 0.51–0.77, *p* = 0.983) (see Figure 2a–c).

The TI cut-offs for myocarditis were calculated by means of the Youden index (cut-off #1: 0.34 (sens.: 78%, spec.: 61%, PPV: 50%, NPV: 84%)) and to depict maximum specificity for myocarditis (cut-off #2: 0.42 (sens.: 48%, spec.: 85%, PPV: 61%, NPV: 76%)).

### 3.4. Binary Logistic Regression Analysis before Propensity Score Weighting

Amongst the investigated echocardiographic parameters, TI was the best predictor for the presence of myocarditis in binary logistic regression analysis (TI: B(SE) = 0.81(0.23), *p* = 0.0004; see Table 3).

### 3.5. Propensity Weighted Logistic Regression Analysis

To account for covariate imbalances between the groups, we further conducted propensity score weighing using GBM. Covariates included were z-score of age, z-score of E/e‘, z-score of EF, diabetes mellitus, hyperlipidemia and arterial hypertension. After weighted binary logistic regression analysis, the association of the TI with the presence of myocarditis remained highly significant (B(SE) = 1.09(0.32), *p* = 0.0009).

## 4. Discussion

The initial diagnostic workup of patients with suspected myocarditis can be quite challenging in clinical practice. As such, patients can present with a wide variety of echocardiographic findings, ranging from an apparently normal TTE exam to LV dilatation with severely impaired systolic function [7,9,10,11].

In previous studies, the TI was identified as a sensitive tool for diagnosis and risk stratification of patients with heart failure [16,23,24], chronic coronary syndrome [19] and acute myocardial infarction [25,26]. However, despite the promising findings of previous studies, evidence on the clinical aspects of the TI in myocarditis is very scarce. To the best of our knowledge, only one study has so far investigated the TI in the context of myocarditis. In their study, Yadav et al. investigated the diagnostic implications of the TI in 67 children with severe dengue fever and suspected asymptomatic myocardial involvement. Intriguingly, the authors reported that an abnormal TI was superior to ejection fraction and E/e’ for the identification of concomitant myocarditis in affected patients [27].

These findings are well in line with the results of our study. Thus, patients with myocarditis in our study had significantly higher, and thus pathological, values of TI (median 0.41 vs. median 0.31, *p* < 0.0001) despite no significant difference in LV systolic function (median 55% vs. median 55%, n.s., see Figure 1) when compared to controls. In contrast, the difference in LV systolic function and TI between ACS patients and controls was comparably modest (each *p* < 0.05). The possible diagnostic ability of the TI for myocarditis became apparent when AUCs for the prediction of myocarditis were calculated in the total cohort. Hence, the observed AUC of the TI was distinctively better than that of EF or E/e‘ (TI: 0.71, *p* < 0.0001, EF: 0.57, *p* = 0.112, E/e‘: 0.64, *p* = 0.983). To complement this finding, we additionally conducted binary logistic regression analysis, where the TI was highly significant for the prediction of myocarditis (B(SE) = 0.81(0.23), *p* = 0.0004). Importantly, this finding even remained statistically significant after the adaption for possible confounders (age, EF, E/e‘, diabetes mellitus, hyperlipidemia and arterial hypertension) using propensity score weighted regression analysis. Notably, the predictive ability of the TI for myocarditis in the total study cohort was not achieved by any of the other conventional echocardiographic measures, thus suggesting that the TI could be a useful adjunctive diagnostic tool in the workup of patients with suspected myocarditis.

Despite the potential diagnostic utility of calculating the TI in patients with chest pain, one has to consider that this echocardiographic measure can merely depict a dysfunction of overall cardiac performance [15]. As such, the TI is very sensitive for functional abnormalities of the LV, yet it is not specific for any myocardial pathology. Consequently, the TI is not diagnostic for myocarditis per se, but it can support the diagnosis in patients who fulfill the current diagnostic criteria of clinically suspected myocarditis issued by the European Society of Cardiology (ESC) [6]. In this regard, the observation that the TI was more pathological in patients with myocarditis than in patients with ACS (median 0.41 vs. 0.35), despite the latter presenting with lower values of EF, could mean that the TI could also complement conventional diagnostic modalities in the differential diagnosis between these two disease entities, probably by use of the calculated cut-offs for TI. This could be of special interest for clinicians since patients with acute myocarditis and ACS frequently present with similar echocardiographic findings, thus posing challenges in the initial diagnostic workup. However, with regard to the low sample size of our cohort, the aforementioned findings of our study need to be confirmed by larger studies in the future.

## 5. Conclusions

Compared to controls, patients with myocarditis had significantly higher values of TI, despite no differences in LV systolic function. In the total study cohort, the TI was superior to EF and E/e’ in its discriminatory ability for myocarditis. Therefore, the TI could be a helpful addition in the initial diagnostic workup of patients with suspected myocarditis, especially when conventional TTE appears to be normal.

## 6. Limitations

Major limitations of the current study are the low sample size and the fact that the study was conducted at a single study center only. Furthermore, the measurement of echocardiographic parameters and calculation of the TI were conducted retrospectively and unblinded. Hence, we cannot exclude a bias on this level. Therefore, large multicenter studies need to address and confirm our findings in the future.

## Figures and Tables

**Table 1:** Baseline characteristics, laboratory parameters and comorbidities of enrolled patients. Abbreviations: ACS = acute coronary syndrome, BMI = body mass index, IQR = interquartile range.

	Control (*n* = 50)		ACS (*n* = 29)		Myocarditis (*n* = 40)		
	Median	IQR	Median	IQR	Median	IQR	*p*-Value
Age (years)	70	62–76	52	49–55	35	25–43	<0.0001
Serum creatinine (mg/dL)	0.88	0.77–0.99	0.97	0.85–1.01	0.89	0.74–1.00	0.110
C-reactive protein (mg/dL)	0.20	0.10–0.43	0.20	0.10–1.00	2.35	0.68–6.18	<0.0001
High sensitivity troponin (hsTnT) (ng/L)	14	11–21	244	80–681	375	135–862	<0.0001
Pro brain natriuretic peptide (pBNP) (ng/L)	1034	254–2881	110	83–240	432	232–1090	0.006
	**%**	***n***	**%**	***n***	**%**	***n***	***p***-**Value**
Male sex	52.0	26	75.9	22	72.5	29	0.049
Diabetes mellitus	18.0	9	27.6	8	2.5	1	0.010
Hyperlipidemia	62.0	31	82.8	24	7.5	3	<0.0001
Obesity (BMI >30 kg/m²)	29.8	14	37.9	11	12.5	5	0.047
Arterial hypertension	80.0	40	65.6	19	10.0	4	<0.0001
History of smoking	50.0	22	55.2	16	17.5	7	0.001

**Table 2:** Echocardiographic parameters. Abbreviations: IQR = interquartile range, IVSDd = interventricular septum thickness in diastole, LVEDd = left ventricular end-diastolic diameter in diastole, TI = Tei index.

	Control (n = 50)		ACS (n = 29)		Myocarditis (n = 40)		
	Median	IQR	Median	IQR	Median	IQR	*p-*Value
Ejection fraction (%)	55	50–60	48	40–55	55	48–60	0.013
IVSDd (mm)	11	10–13	12	10–13	11	9–13	0.302
LVEDd (mm)	50	43–55	47	42–50	48	44–51	0.119
E/e‘	11.3	8.3–14.1	10.0	8.1–11.9	8.6	7.0–10.0	0.041
TI	0.31	0.23–0.36	0.35	0.30–0.44	0.41	0.35–0.48	<0.0001

**Table 3:** Binary logistic regression analysis of echocardiographic parameters for presence of myocarditis in the total cohort before propensity weighting. Abbreviations: B = regression coefficient, EF = ejection fraction, SE = standard error, TI = Tei index.

	B	SE	*p*-Value
TI, z-score	0.81	0.23	0.0004
EF, z-score	0.14	0.21	0.498
E/e‘, z-score	−0.22	0.27	0.416

## Data Availability

The data underlying this article will be shared on reasonable request to the corresponding author.
